# Landscape of cardiometabolic risk factors in Chinese population: a narrative review

**DOI:** 10.1186/s12933-022-01551-3

**Published:** 2022-06-21

**Authors:** Jian-Jun Li, Hui-Hui Liu, Sha Li

**Affiliations:** Cardiometabolic Center, State Key Laboratory of Cardiovascular Disease, National Center for Cardiovascular Diseases, Fuwai Hospital, Chinese Academy of Medical Sciences and Peking Union Medical College, No.167 BeiLiShi Road, XiCheng District, Beijing, 100037 China

**Keywords:** Cardiometabolic risk factors, Cardiovascular Disease, China, Review

## Abstract

With rapid economic growth and changes at all levels (including environmental, social, individual), China is facing a cardiovascular disease (CVD) crisis. In China, more than 40% of deaths are attributable to CVDs, and the number of CVD deaths has almost doubled in the past decades, in contrast to a decline in high-income countries. The increasing prevalence of cardiometabolic risk factors underlies the rise of CVDs, and thus curbing the rising cardiometabolic pandemic is imperative. Few articles have addressed this topic and provided an updated review of the epidemiology of cardiometabolic risk factors in China.

In this narrative review, we describe the temporal changes in the prevalence of cardiometabolic risk factors in the past decades and their management in China, including both the well-recognized risk factors (general obesity, central obesity, diabetes, prediabetes, dyslipidemia, hypertension) and the less recognized ones (hyperhomocysteinemia, hyperuricemia, and high C-reactive protein). We also summarize findings from landmark clinical trials regarding effective interventions and treatments for cardiometabolic risk factors. Finally, we propose strategies and approaches to tackle the rising pandemic of cardiometabolic risk factors in China. We hope that this review will raise awareness of cardiometabolic risk factors not only in Chinese population but also global visibility, which may help to prevent cardiovascular risk.

## Introduction

**Fig. 1:**
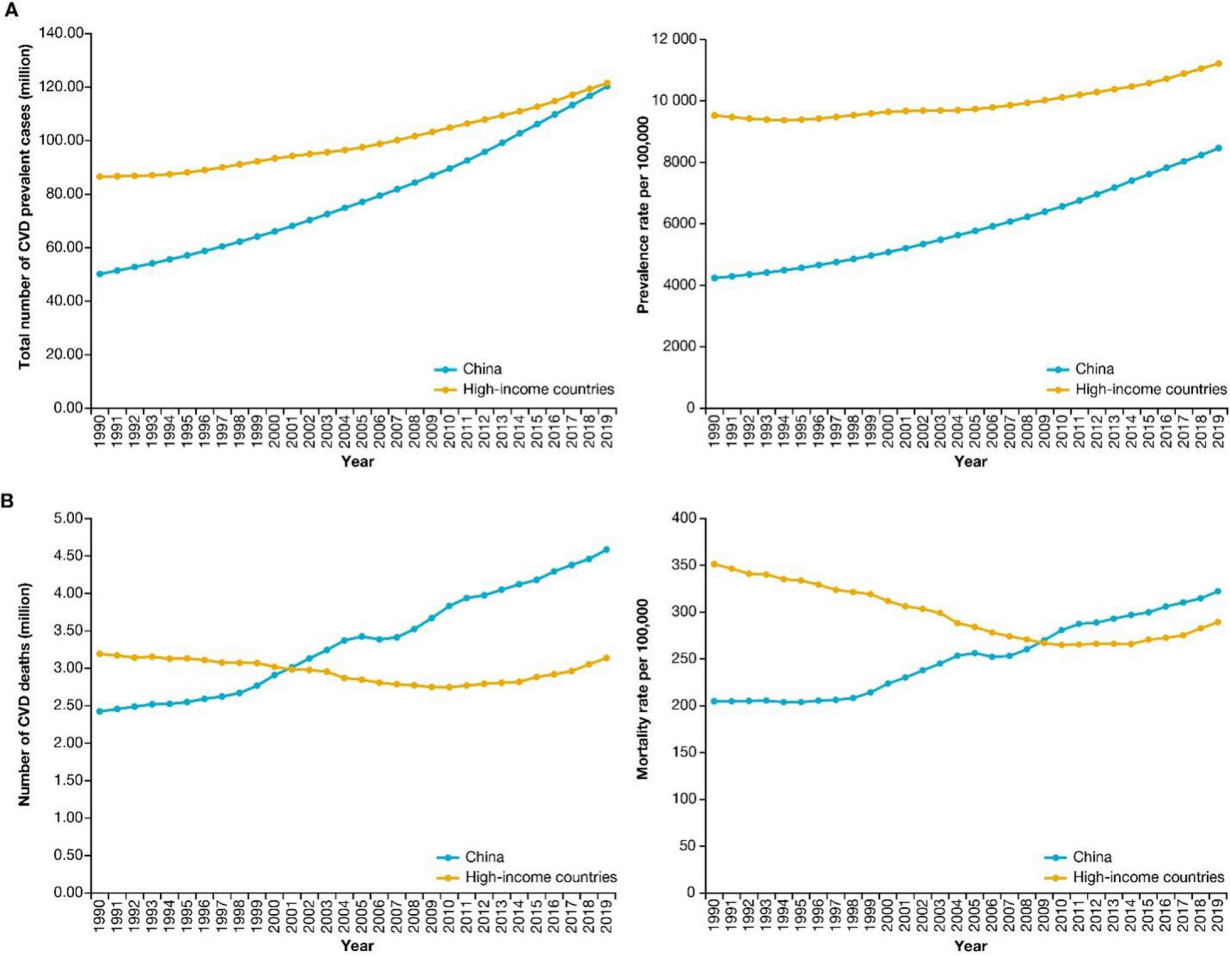
Prevalence and mortality of cardiovascular diseases in China and high-income countries from 1990 to 2019. **A** Number (left panel) and rate (right panel) of CVD prevalent cases in China and high-income countries; **B **Numebr of CVD deaths (left panel) and mortality rate (right panel) in China and high-income countries. Figures were developed according to data from http://ghdx.healthdata.org/gbd-results-tool

China is facing a cardiovascular disease (CVD) crisis. In China, more than 40% of deaths are attributable to CVD [1], and the total number of CVD deaths in China is now larger than any other country around the world [2]. Notably, the number of prevalent cases of CVD has more than doubled from 50.13 million in 1990 to 120.33 million in 2019, and the number of deaths attributed to CVD almost doubled from 2.42 million in 1990 to 4.58 million in 2019 [3]. The increasing trend of CVD mortality in China is in stark contrast to a decline of that in high-income countries since the late twentieth century (Fig. 1). More alarming, if we raise the bar and look into how many have ideal cardiovascular health, it is sobering to see that only 1% of the Chinese adult population without a history of CVD meet the criteria [4].

To combat this public health crisis, we need to address the root causes and take a holistic view. In this article, we adopt the concept of cardiometabolic risk to encompass a broad spectrum of interrelated risk factors that are associated with a life-long risk for CVDs [5]. This concept is in line with the new medicine specialty, cardiometabolic medicine, which is a multidisciplinary and integrated model for preventing and treating CVDs. We outline the prevalence of cardiometabolic risk factors and the status of disease management in China and compare them with those in the United States (US). We also summarize high-quality evidence from important clinical trials in China that informed the intervention and treatment of cardiometabolic risk factors. Finally, we propose strategies and approaches at national and organizational levels to tackle the rising pandemic of cardiometabolic risk factors. The review will call to attention of cardiometabolic risk factors not only in Chinese population but also global visibility, which may help to the prevention of cardiovascular risk.

## Methods

**Fig. 2:**
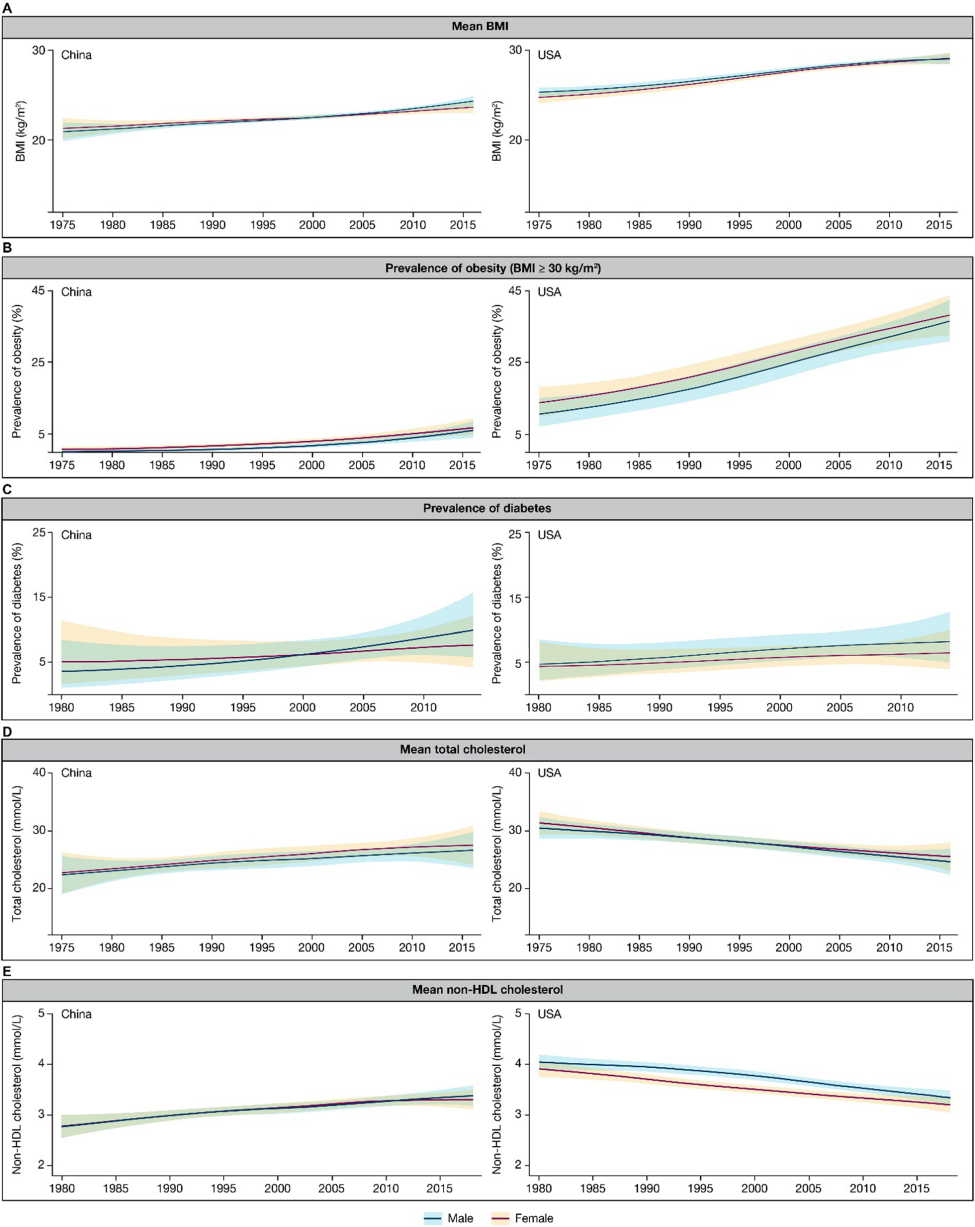

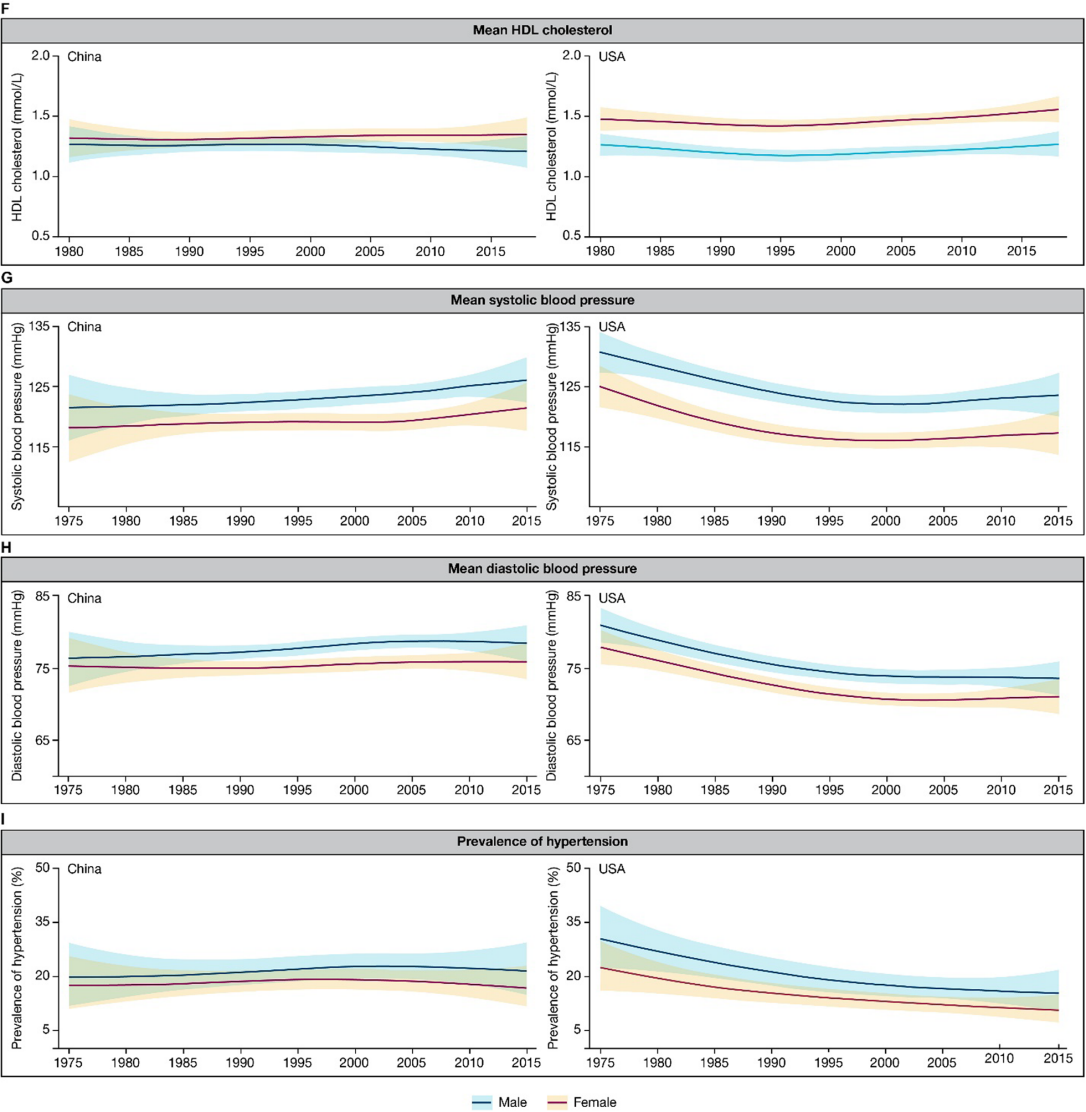
Trend in the prevalence and mean value of conventional cardiometabolic risk factors in China and the US. Temporal treads in the mean values or prevalence of cardiometabolic risk factor among male and female adults in China (left panel) and the United States (right panel). Adapted from https://www.ncdrisc.org/index.html

We performed a narrative review of the literature in the PubMed database. We entered search terms for individual cardiometabolic risk factor and “China” or “Chinese” in the titles or abstracts. English-language publications were screened, and reports of large-scale cohort studies that enrolled a nationally representative sample of subjects with the use of multistage, stratified, random sampling methods are cited as the primary data source in this review (Table 1). We also cited data from the Global Burden of Diseases, Injuries, and Risk Factors Study (GBD) and Non-Communicable Disease (NCD) Risk Factor Collaboration global databases, which provide standardized consecutive data from different countries; temporal curves of mean values and prevalence of cardiometabolic risk factors in China and the US according to these databases are presented in Fig. 2. Data from the National Health and Nutrition Examination Survey are cited for the prevalence and management in the US. In addition, data from meta-analyses and studies of limited sample sizes are used where large-scale nationwide data in China are limited, especially for the less recognized risk factors. Furthermore, we searched for clinical trials of interventions or treatments for cardiometabolic risk factors in China; results from well-designed, adequately powered randomized controlled trials are summarized (Table 2).

**Table 1:** Large-scale cohort studies of cardiometabolic risk factors in China

Cohort	Years of study	Geographic coverage	Sample size^a^	Age
China Health and Retirement Longitudinal Study	2011–2018	150 county-level units from 28 provinces	17 500	≥ 45 years
InterAsia	2000–2001	20 primary sampling unites covering 10 provinces	15 838	35–74 years
China National Nutrition Surveys/China Health and Nutrition Survey/China National Nutrition and Health Surveillance	1959–2015	31 provinces, autonomous regions, and municipalities	44 097	All ages
China Noncommunicable Disease Surveillance 2010	2010	162 study sites covering 31 provinces, autonomous regions, and municipalities	98 658	≥ 18 years
China Kadoorie Biobank	2004–2014	Five urban (Qingdao, Harbin, Haikou, Suzhou, Liuzhou) and 5 rural (Gansu, Sichuan, Henan, Zhejiang, Hunan) areas	512 891	30–79
China Chronic Disease and Risk Factors Surveillance study	2004–2018	31 provinces, autonomous regions, and municipalities	746 020	≥ 18 years
China National Diabetes and Metabolic Disorders Study	2007–2008	152 urban street districts and 112 rural villages	54 240	≥ 20 years
Thyroid Disorders, Iodine Status and Diabetes Epidemiological Survey	2015–2017	31 provinces, autonomous regions, and municipalities	75 880	≥ 18 years
China Multi-Ethnic Cohort	2018–2019	Participants from six ethnic minority groups and the Han group were enrolled in Southwest China	99 556	30–79
China Patient-centered Evaluative Assessment of Cardiac Events Million Persons Project	2014–2019	3041 primary care institutions covering 31 provinces	2 660 666	35–75 years
China Cardiometabolic Disease and Cancer Cohort Study	2010–2016	20 communities covering 16 provinces, autonomous regions, or municipalities of mainland China	193 846	≥ 40 years
China National Stroke Screening and Prevention Project	2014–2015	200 project areas covering 31 provinces, autonomous regions, or municipalities of mainland China	726 451	≥ 40 years
China Hypertension Survey	2012–2015	31 provinces, autonomous regions, or municipalities of mainland China	487 349	≥ 15 years

^a^If sample size differs between rounds of study, the maximum sample size is provided

**Table 2:** Landmark clincial trials of cardiometabolic risk factors in China

Study	Year	Participants	Intervention or Treatment	Outcomes (Intervention/Treatment vs Control)
Da Qing Diabetes Prevention Study [69]	1986–2016	577 adults aged 25–74 with impaired glucose tolerance	Lifestyle intervention groups (diet and/or exercise) *vs*. no intervention	Primary outcomes: CVD events, HR = 0.74 (95% CI = 0.59, 0.92)
				Microvascular complications, HR = 0.65 (95% CI = 0.45, 0.95)
				CVD mortality, HR = 0.67 (95% CI = 0.48, 0.94)
				All-cause mortality, HR = 0.74 (95% CI = 0.61, 0.89)
				Secondary outcomes: Stroke, HR = 0.75 (95% CI = 0.59, 0.96)
				Coronary heart disease, HR = 0.73 (95% CI = 0.51, 1.04)
				Hospital admission for heart failure, HR = 0.71 (95% CI = 0.48, 1.04)
				Diabetes, HR = 0.61 (95% CI = 0.45, 0.83)
				Retinopathy, HR = 0.60 (95% CI = 0.38, 0.95)
				Nephropathy, HR = 0.68 (95% CI = 0.36, 1.28)
				Neuropathy, HR = 0.57 (95% CI = 0.24, 1.36)
China Stroke Primary Prevention Trial (NCT00794885) [70]	2008–2013	20 702 adults aged 45–75 years with hypertension and without a history of CVDs	Folic acid plus enalapril *vs*. enalapril	Primary outcome: Stroke, HR = 0.79 (95% CI = 0.68, 0.93)
				Secondary outcomes: CVD events, HR = 0.80 (95% CI = 0.69, 0.92)
				Ischemic stroke, HR = 0.76 (95% CI = 0.64, 0.91)
				Hemorrhagic stroke, HR = 0.93 (95% CI = 0.65, 1.34)
				MI, HR = 1.04 (95% CI = 0.60, 1.82)
				All-cause mortality, HR = 0.94 (95% CI = 0.81, 1.10)
China Salt Substitute and Stroke Study (NCT02092090) [71]	2014–2020	20 995 adults who had a history of stroke or were aged ≥ 60 years and had hypertension	Salt substitute *vs*. regular salt	Primary outcome: Stroke, rate ratio = 0.86 (95% CI = 0.77, 0.96)
				Secondary outcomes: Major CVD events, rate ratio = 0.87 (95% CI = 0.80, 0.94)
				All-cause mortality, rate ratio = 0.88 (95% CI = 0.82, 0.95)
Chinese Coronary Secondary Prevention Study [72]	1996–2003	4 870 adults aged 18–70 years with a history of MI	Xuezhikang *vs*. placebo	Primary outcome: Major coronary events, relative risk, 0.55
				Secondary outcomes: CVD morality, relative risk = 0.70 (95% CI = 0.54, 0.89)
				All-cause mortality, relative risk = 0.67 (95% CI, 0.52, 0.82)
				Coronary revascularization, relative risk = 0.64 (95% CI = 0.47, 0.86)
				Change in lipoprotein lipids,− 10.9% for total cholesterol,− 17.6% for LDL cholesterol,− 16.6% for non-HDL cholesterol,− 14.6% for triglycerides, and 4.2% for HDL cholesterol
Strategy of Blood Pressure Intervention in the Elderly Hypertensive Patients (NCT03015311) [73]	2017–2020	8511 patients aged 60–80 years with hypertension	Intensive treatment (a systolic blood-pressure target of 110 to less than 130 mm Hg) *vs*. standard treatment (a target of 130 to less than 150 mm Hg)	Primary outcome: CVD events, HR = 0.74 (95% CI = 0.60, 0.92)
				Secondary outcomes: Stroke, HR = 0.67 (95% CI = 0.47, 0.97)
				Acute coronary syndrome, HR = 0.67 (95% CI = 0.47, 0.94)
				Acute decompensated heart failure, HR = 0.27 (95% CI = 0.08, 0.98)
				Coronary revascularization, HR = 0.69 (95% CI = 0.40, 1.18)
				Atrial fibrillation, HR = 0.96 (95% CI = 0.55, 1.68)
				CVD mortality, HR = 0.72 (95% CI = 0.39, 1.32)
Acarbose Cardiovascular Evaluation (NCT00829660) [74]	2009–2015	6522 patients with coronary heart disease and impaired glucose tolerance	Acarbose *vs*. placebo	Primary outcome: CVD events, HR = 0.98 (95% CI = 0.86, 1.11)
				Secondary outcomes: CVD events, all-cause mortality, CVD mortality, impaired renal function, not significantly different between arms
				Diabetes, rate ratio = 0.82 (95% CI = 0.71, 0.94)

*CI* confidence interval, *CVD*  cardiovascular disease, *HR*  hazard ratio, *MI* myocardial infarction

### Cardiometabolic risk factor definition, interaction, and contribution to CVDs

**Fig. 3:**
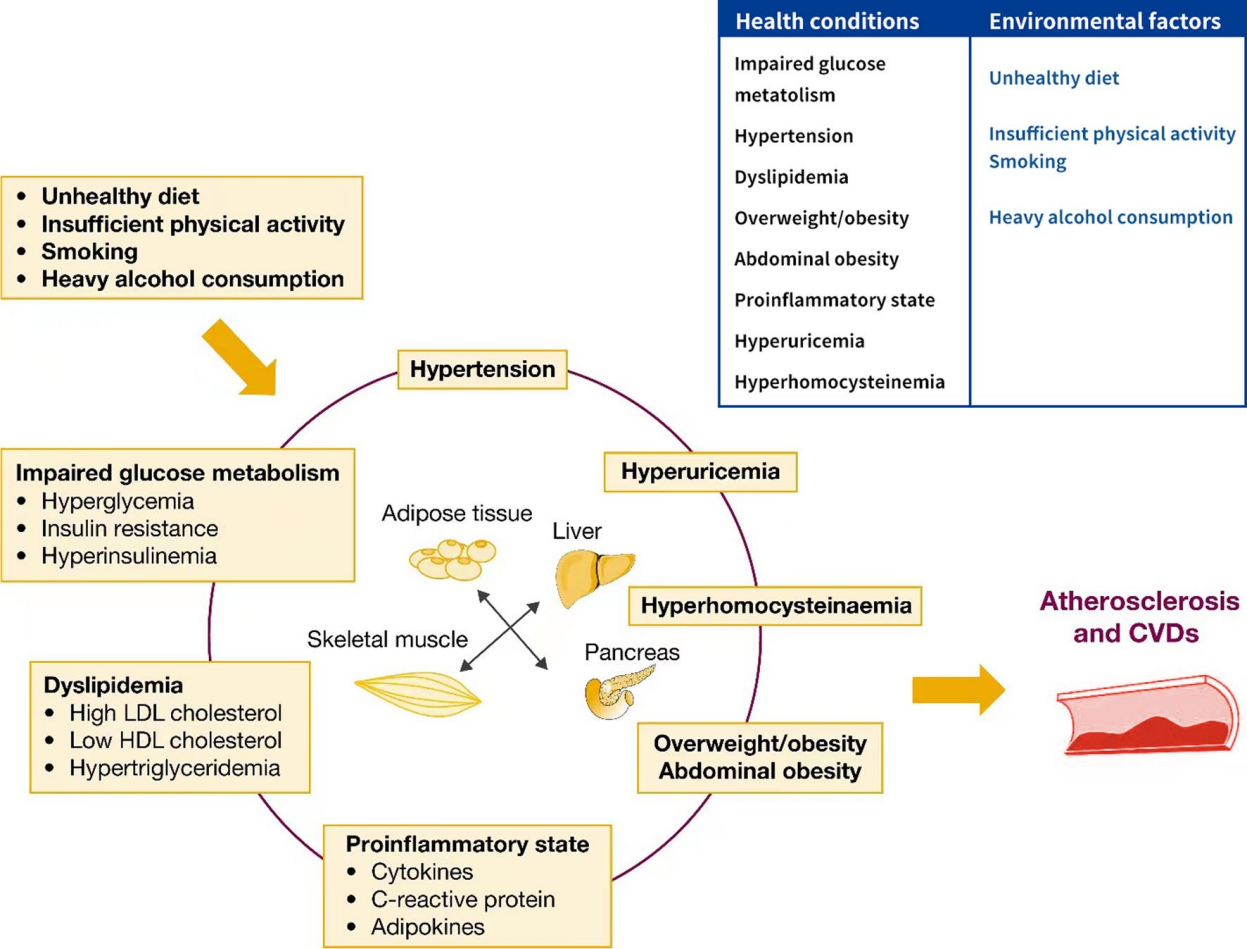
An interactive network of cardiometabolic risk factors. Cardiometabolic risk factors include health conditions, biomarkers, and environmental factors. The heath conditions, including overweight or obesity, abdominal obesity, impaired glucose metabolism, dyslipidemia, and hypertension, frequently cluster in individuals at risk for CVDs and may contribute to each other. Underlying this phenomenon is a close link between different organs and tissues. At the molecular level, these health conditions are usually accompanied by an increase in biomarkers such as cytokines, C-reactive protein, adipokines, uric acid, and homocysteine in the blood stream; the increase in these biomarkers represent a proinflammatory state and oxidative stress, which contribute to atherosclerosis and development of CVDs. CVD, cardiovascular disease; HDL, high-density lipoprotein; LDL, low-density lipoprotein

The first official use of the term “cardiometabolic risk” can be dated back to 2006 in a joint scientific statement from the American Heart Association and American Diabetes Association, intended to denote a spectrum of risk factors for diabetes and CVDs [6]. This concept was further reframed by the Cardiometabolic Risk Working Group as an umbrella term to encompass a comprehensive list of factors that contribute to CVDs and diabetes [5]. We present the cardiometabolic risk factors that have been recognized to date and their interactions in Fig. 3. This list includes not only the well-recognized risk factors commonly used for CVD risk assessment but also the newly emerging factors involved in the pathogenesis of and association with CVDs in population studies. Concurrent existence of cardiometabolic risk factors is common [7, 8]; while each cardiometabolic risk factor poses increased risk for CVDs, the presence of comorbidities substantially elevates the risk [7–9]. The theoretical foundation of cardiometabolic risk is that an interactive constellation of health conditions, which are modifiable by behavioral factors, play critical roles in the pathogenesis of CVDs [5]. Impaired glucose metabolism, dyslipidemia, and hypertension can contribute to each other, and overweight or obese individuals, especially those with abdominal obesity, are at high risk for metabolic abnormalities [5, 10]. At the molecular level, a hallmark of these health conditions is a concomitant increase in proinflammatory molecules (represented by C-reactive protein), uric acid, and homocysteine, which may result in metabolic dysregulation, systemic inflammation, oxidative stress, and ultimately atherosclerosis and CVDs [10–13].

### Prevalence and management of cardiometabolic risk factors in China

#### General obesity and abdominal obesity

China has experienced a marked increase in overweight and obesity in the past decades. According to the Chinese body mass index (BMI) cutoffs (overweight: BMI of ≥ 24 kg/m^2^ and < 28 kg/m^2^; obesity: BMI ≥ 28 kg/m^2^) [14], the prevalence of overweight and obesity among adults (≥ 18 years) increased substantially, which was 16.4%, 22.8%, and 34.3% for overweight and 3.6%, 7.1%, and 16.4% for obesity in 1992, 2002, and 2015–2019, respectively [15]. The rising trend of overweight and obesity is consistent if defined using the World Health Organization international criteria (overweight: BMI of ≥ 25 kg/m^2^ and < 30 kg/m^2^; obesity: BMI ≥ 30 kg/m^2^); the prevalence of overweight and obesity rose from 7.9% and 0.2% to 31.3% and 5.3% in men and from 9.6% and 0.8% to 24.9% and 6.3% in women from 1975 to 2014, respectively (Fig. 2B) [16, 17]. The cases of obesity among Chinese adults are predominantly moderate, with a low rate of severe (0.6% in men and 1% in women in 2016) and morbid obesity (0.2% in both genders in 2016) [16]. Obesity used to be more prevalent in the urban than rural areas, but the gap has gradually closed, and now rural areas face the same obesity epidemic as the urban areas [18, 19]. Overweight or obesity is more and more prevalent as age increases but the prevalence declines slightly in those aged ≥ 60 years [9, 19]. However, young adults (aged 18–44 years), especially young men, experienced the greatest increase in the prevalence of overweight or obesity between 1992 and 2002 [20]. The trend that Chinese are getting fatter is also reflected by the gradual increase in mean BMI, from 20.9 kg/m^2^ in men and 21.3 kg/m^2^ in women in 1975, 22.2 kg/m^2^ and 22.3 kg/m^2^ in 1995, to 24.3 kg/m^2^ and 23.6 kg/m^2^ in 2016 (Fig. 2A) [16]. It is estimated that every year mean BMI in the Chinese population increased by 0.17 kg/m^2^ between 2004 and 2010, which slowed down to 0.09 kg/m^2^ between 2010 and 2018 [21]. Similarly, the US has also witnessed increasingly higher BMI in the population; the mean BMI increased from 25.3 kg/m^2^ to 29.0 kg/m^2^ in men and from 24.7 kg/m^2^ to 29.1 kg/m^2^ in women between 1975 and 2016 (Fig. 2A) [16]. Obesity increased from 10.7 to 36.5% in men and from 13.8% to 38.2% in women between 1975 and 2016 in the US (Fig. 2B), skewing toward severe and morbid obesity [16]. However, if ranked by the total number of prevalent cases, China replaced the US as the epicenter with the largest number of obese people in 2014 [17].

BMI indicates little about body composition. As a complement to BMI, waist circumference is used as a surrogate for abdominal adiposity, which may be a better predictor for metabolic abnormalities compared with BMI [22, 23]. The mean waist circumference of Chinese adults is becoming larger and larger, from 76.0 cm in 1993, 80.7 cm in 2007, to 83.4–83.5 cm in 2015–2017 [19, 24]. Abdominal obesity in China (defined according to the International Diabetes Federation criteria as a waist circumference of ≥ 90 cm for males and ≥ 80 for females among the Chinese population [25]) increased from 20.2% in 1993, 25.9% in 2007, to 35.4%–46.9% in 2015–2017 [19, 24]. Between 2007 and 2017, the most substantial rise in abdominal obesity took place in the rural areas, which overtook the urban areas in terms of prevalence by 2017 [19]. Likewise, the waist circumference of the US population also increased, from 98.6 cm and 92.2 cm in 1999–2000 to 102.3 cm and 98.4 cm in 2015–2016 for men and women, respectively [26]; across both genders, 54.5% had abdominal obesity in 2011–2012, which increased to 59.1% in 2017–2018 [27].

#### Diabetes and prediabetes

The prevalence of diabetes in China has increased by more than tenfold since the 1980s. In 1980, 0.67% of adults in China had diabetes, whereas the prevalence increased to 10.9–12.8% from 2010 to 2018 according to different studies, although the estimates were not directly comparable due to differences in diagnostic criteria and sampling methods [28–31]. As the total number of individuals with diabetes has exceeded that in any other country, China has emerged as an epicenter for diabetes [32]. Prediabetes, a precursor to diabetes, has also shown a strikingly rising trend among Chinese adults, the prevalence was 15.5% in 2007–2008, 35.7% in 2013, and 38.1% in 2018 [29–31, 33]. Diabetes was more common in men, senior individuals, urban areas, and economically developed areas than their counterparts [28, 29]. In the US, the trajectory of increasing diabetes prevalence is similar to that in China in the past decades (Fig. 2C) [16].

The unique characteristics of Chinese patients with diabetes include young age of onset and a low BMI. Young-onset diabetes, defined as a diagnosis before 40 years of age, is common in Asia, which composes approximately one-fifth of diabetes cases [34]. The young-onset patients may experience more rapid deterioration of β-cell function and are predisposed to life-long complications [35]. Asian patients with diabetes have a lower mean BMI than those in the US [36], and the positive linear relationship between BMI and risk for diabetes extends below a BMI of 25 [37]. Moreover, impaired glucose tolerance is a more common presentation compared with impaired fasting glucose [33].

The gaps between China and the US with regard to the awareness and management of diabetes are substantial. In 2010 about two thirds of individuals with diabetes were undiagnosed in China; 25.8% of patients received antidiabetic medications, and among them, 39.7% achieved the glycemic control target [28]. The situation of diabetes management was still disturbing in 2013 and 2018: 36.5% were aware, 32.2% were treated, and 49.2% of treated patients achieved the target in 2013 [29], and the corresponding rates in 2018 were 36.7%, 32.9%, and 50.1% [30]. The inadequacy in diabetes diagnosis and management was particularly worse in the rural areas and in economically underdeveloped regions [28]. In comparison, the management of diabetes is better in the US, with the rates of awareness, treatment, and glycemic control being 86.8%, 82.5%, and 58.8%, respectively, according to a systemic review of data up to 2014 [38]. The inadequate diabetic care in China is likely the reason why diabetes continues to contribute significantly to excess mortality and poses a greater health threat compared with that in high-income countries [39, 40].

#### Dyslipidemia

Population-based data on the lipid profile for the Chinese population were limited before the twenty-first century. The nationwide study InterASIA in 2000–2001 provided the first glimpse into the prevalence of dyslipidemia in China, which found that 53.6% of adults aged 35–74 years had dyslipidemia, and in regard to its components, 9.0%, 5.1%, and 19.2% had high total cholesterol, high low-density lipoprotein cholesterol (LDL-C), and low high-density lipoprotein cholesterol (HDL-C), respectively [7, 41]. Studies in 2014–2019 revealed a consistently high prevalence of dyslipidemia in China, ranging from 33.8 to 43% among adults of middle or senior ages; presentations with high triglycerides (16.9–22.4%) and low HDL-C (15.6–19.9%) were more common compared with high total cholesterol (7.1%–11.3%) and high LDL-C (4.0–8.1%) [42–44]. Among adults aged ≥ 18 years, the prevalence of high total cholesterol, high triglycerides, high LDL-C, and low HDL-C was reported to be 6.9%, 13.8%, 8.1%, and 20.4% in 2013–2014, respectively [45]. Adults aged ≥ 50 are more likely to have dyslipidemia, while the gender difference is disputable [42, 44]. Notably, China is ranked as one of the top countries with the largest magnitudes of increase in mean non-HDL-C [46]. Mean total cholesterol increased from 4.1 mmol/L in both Chinese men and women in 1980 to 4.6 mmol/L and 4.7 mmol/L in 2018, driven by the increase in mean non-HDL-C, while the mean level of HDL-C remained relatively static (Fig. 2D, E and F) [16]. The increasing trend of mean cholesterol levels in China contrasts with the dramatic declines in the US; the mean total cholesterol in the US decreased from 5.3 mmol/L in males and 5.4 mmol/L in females in 1980 to 4.6 mmol/L and 4.7 mmol/L, respectively, in 2018, with a consistent reduction in mean non-HDL-C (Fig. 2D and E) [16]. 

There is a paucity of data on the epidemiology of familial hypercholesterolemia (FH) in China. It is estimated that 8% of FH cases worldwide are in China, with 2 765 420–6 913 550 cases of heterozygous FH (HeFH) and 2205–4609 cases of homozygous FH (HoFH), falling at a prevalence within 1/200–1/500 and 1/300 000–1/600 000, respectively [47]. Based on the China Acute Myocardial Infarction Registry, about 4.2% of patients with acute myocardial infarction (MI) had heterozygous FH [48]. One study involving 8050 patients undergoing coronary angiography found that 3.5% of them were identified to have FH phenotypes [49], and another study including 1843 patients with MI who received coronary angiography showed that 3.9% had FH [50]. Less than 1% of Chinese patients with FH were diagnosed or treated before 2018 because there were no standard diagnosis criteria for FH in China until 2018 and Chinese patients usually present with lower levels of cholesterol than thresholds based on the western population [47, 51]. Among Chinese patients with FH, mutations in LDL receptor are most common (82%), followed by mutations in apolipoprotein B (9%) [52]. The underdiagnosis of FH (< 10%) is also an issue in most high-income countries including the US where genetic testing is not widely used [53].

Yet the increasing prevalence of dyslipidemia has not received due attention or action in China. In 2000–2001, 8.8% and 7.5% of men and women, respectively, who had dyslipidemia in China were aware of the condition, 3.5% and 3.4% were treated, and 1.9% and 1.5% achieved a good control of total cholesterol [41]. The situation during 2011–2019, though slightly improved since 2000 was still far from satisfactory; the rate of awareness ranged from 19.6 to 64.0%, and that of treatment ranged from 13.2 to 39.3% overall and 5.5–42.1% in those at high risk for atherosclerotic CVDs in different studies; the rate of good control ranged from 4.6 to 25.8% overall and 19.9–42.9% in the high-risk group [42, 44, 45, 54, 55]. Statins constitute the mainstream medications used for dyslipidemia, 94.5% of treated patients received statins, including 42.5% treated with atorvastatin, 29.0% with simvastatin, and 15.2% with rosuvastatin as shown in a study by Gao et al. [55]. However, statins and other lipid-lowering medications are not readily available in primary care settings in China, as only 49.7% of primary care institutes had stocked statins and 10.2% had Xuezhikang, limiting their widespread use for dyslipidemia management [42]. Compared with China, the diagnosis and treatment rates in the US are higher; in the US, 73.3% of individuals with dyslipidemia were aware of the condition, 54.1% received lipid-lowering medications, and 35.7% achieved good control between 2011 and 2012, as opposed to 19.6%, 13.2%, and 4.6% in China during this time frame [54].

#### Hypertension

Hypertension is the top cardiometabolic risk factor accounting for the largest cardiovascular burden [2]. In China, the prevalence of hypertension (defined as a systolic blood pressure [SBP] of ≥ 140 mm Hg and a diastolic blood pressure [DBP] of ≥ 90 mm Hg, as well as taking antihypertensive medications in some studies) increased from 15.3 to 15.7% in 1991 to 23.2–25.6% in 2012–2015 among adults in different studies [19, 56–58]. The proportion of adults with prehypertension (defined as an SBP of 120–139 mm Hg and a DBP of 80–89 mm Hg) also increased, from 30.1% in 1991 to 43.1% in 2015 [58]. Rural areas have experienced the greatest increase, which had a lower prevalence of hypertension than the urban areas in 2007 but the difference reverted in 2017 [19, 57]. Hypertension is more common in men than women, and the prevalence increases with age [19, 56]; however, the faction of young adults with hypertension is also alarming, which was 10.3% among those aged 18–39 years and 28.7% for 40–59 years in 2015, as opposed to 47.1% for ≥ 60 years. [58]. According to the NCD Risk Factor Collaboration database, the prevalence of hypertension, mean SBP, and mean DBP in China all demonstrated an upward trend from 1975 to 2015; these are opposed to the evident decrease in these metrics in the US (Fig. 2G,H and I) [16]. The prevalence of hypertension decreased from 30.4% in men and 22.5% in women in 1975 to 15.3% and 10.5%, respectively, in 2015 in the US [16].

Despite the alarming trend of rising hypertension, the rates of awareness and treatment remain low in China. During 2011–2017, 27.2–56.1% of patients with hypertension were aware, 22.9–46.8% were treated, and 5.7–20.3% had achieved good control as reported by different studies [54, 56, 57, 59]. Although treatment guidelines recommend using combinations of antihypertensive medications to achieve the control target [60], only 18.5–31.7% received 2 or more antihypertensive medications, and calcium channel blockers were the most frequently prescribed class of medications [56, 59]. The management of hypertension in the US was much better than that in China in 2011–2012, as 84.2% versus 56.1% were aware, 77.9% versus 46.8% were treated, and 54.7% versus 20.3% achieved hypertension control [54]. In 2019, the gaps with regard to awareness, treatment, and control of hypertension in China versus the US were still large, which were 56.4% versus 82.8%, 44.6% versus 73.3%, and 17.8% versus 51.0% [61].

#### Hyperhomocysteinemia, hyperuricemia, and high C-reactive protein

Hyperhomocysteinemia (defined as a serum homocysteine level of > 15 μmol/L) has received attention as a newly emerging risk factor for CVDs [62]. In China, the prevalence of hyperhomocysteinemia increased from 22.7% in 1990–2005, 29.6% in 2006–2012, to 37.2% in 2014–2021 according to 2 studies [62, 63]. The prevalence of hyperhomocysteinemia increases with age, and it is more common in men, rural residents, and the north than their counterparts in China [62, 63]. In comparison, 6.9% of the US population had hyperhomocysteinemia between 2001 and 2006, which was lower than that in China [64].

Hyperuricemia (defined as a serum uric acid level of > 7.0 mg/dL for men and > 5.7 mg/dL for women) is increasingly recognized as a risk factor for CVDs. The prevalence of hyperuricemia in China was estimated to be 8.5% in 2001, which elevated to 18.4% in 2017 [65]. Serum level of uric acid is higher in men than women and increases with age [66]. In comparison, there were no apparent changes in the prevalence of hyperuricemia in the US between 2007 and 2016; the prevalence was 21.4% in 2007–2008 and 20.1% in 2015–2016 [67].

The trend in high C-reactive protein (> 3.0 mg/L and ≤ 10 mg/L) among the Chinese population in past decades is less understood. In 2009, 21.3% of people in China had high C-reactive protein according to the China Health and Nutrition Survey [68]. The mean C-reactive protein level in the Chinese population was lower than that for the US population in 2011–2012 [54].

### Important clinical trials conducted in China and their main findings

Against the backdrop of a rising pandemic and growing attention to cardiometabolic risk factors, a number of clinical trials for risk factor management and CVD prevention were conducted in China in the past decades. We listed the landmark high-quality clinical trials in Table 2. These trials inform clinical practice, patient care, and public health policies regarding the prevention and/or management of cardiometabolic risk factors and CVDs in the Chinese population.

Among these trials, the Da Qing Diabetes Prevention Study is the first randomized controlled trial that provides robust evidence on the benefits of lifestyle intervention in preventing CVDs among individuals with impaired glucose tolerance [69]. The China Stroke Primary Prevention Trial is a renowned study that lends support to folic acid supplementation for preventing CVDs [70]. The effect of salt substitute on CVD prevention was demonstrated by the China Salt Substitute and Stroke Study [71], which presents a cost-effective and scalable approach and may be considered in public health policies. The Chinese Coronary Secondary Prevention Study found that Xuezhikang, an extract of red yeast rice, resulted in significant reductions in CVD endpoints and mortality as well as improvements in lipid profile compared with placebo in patients with a history of MI [72]. This study is the first randomized controlled trial for CVD risk reduction and the only one demonstrating CVD benefits among the Chinese high-risk population.

## Perspectives

The unprecedented rapid pace of economic growth, industrialization, and urbanization in China in the past decades has brought about dramatic changes at economic, environmental, social, and individual levels. Increase in per capita income, surplus food supply, international trade, easy access to and advertisement of unhealthy, high-calorie food and beverages, environments, and occupations, and leisure activities that promote a sedentary lifestyle, social norms, and an aging population altogether underlie the rising prevalence of cardiometabolic risk factors. The threat of a cardiometabolic pandemic is imminent across all ages; in particular, once thought to be chronic diseases that pose a high risk to the elderly, adults under 60 years of age are now also facing a great threat from cardiometabolic risk factors. The risk factors have diversified and are no longer limited to the commonly seen metabolic abnormalities; escalations in important biomarkers (e.g., homocysteine, uric acid, and C-reactive protein) among the Chinese population have drawn increasing attention. Low awareness, inadequate diagnosis, prevention, and treatment, uneven access to health care and medications have hampered proper actions to halt the rising pandemic across China.

The roadmap to curbing the rising pandemic of cardiometabolic risk factors is through concerted efforts at all levels and sectors. First, at the national level, government policy making, implementation, and resource allocation are critical. The Chinese government has integrated a “health” vision into the 14th Five-Year Plan and released a series of public health policies and plans, such as Healthy China 2030, Healthy China Initiative (2019–2030), tobacco control regulations, and smoking bans at public places. Great efforts have been made to achieve “healthy China,” through education and campaigns advocating a healthy lifestyle, set-up of fitness centers and equipment, investment in and reform of the health system, and talent recruitment and professional training to ensure adequate staffing for health care, among others. Second, at the organizational level, structural changes in the health care system are needed. Establishing a multidisciplinary system with good cooperation and coordination from different specialties is imperative given the coexistence of and close link between cardiometabolic risk factors. This need has prompted the formation of cardiometabolic medicine organizations by health care professionals and top-tier hospitals, for instance, the Cardiometabolic Medicine Group of Chinese Medical Doctor Association, the Cardiometabolic Medicine Group of Chinese Society of Cardiology, and the National Center of Cardiometabolic Diseases at Fuwai Hospital. Moreover, measures to promote better disease prevention and management should be implemented at all 3 tiers of hospitals and communities, which requires adequate personnel, resources, and education. Third, there is a dire need for the government to cooperate with the industry to promote the supply and advertisement of healthy food, discourage the consumption of unhealthy food, promote regular physical activities at workplaces and during leisure time, further educate on the hazards of tobacco use, and implement strict smoking bans at public places. Forth, nationwide, large-scale, perspective cohort studies with long-term follow-up and clinical trials in China are still needed to better understand the risk factors, disease course, and outcomes for these cardiometabolic risk factors, especially the less recognized ones, and provide effective intervention and treatment options for the Chinese population. We call for greater attention and prompt action from government, industry, health care institutions, professionals, researchers, and individuals in China to better prevent and manage the cardiometabolic risk factors and ultimately reduce the risk of CVDs.

## Data Availability

Not applicable.
